# Evaluation of the analytical performance of anti-SARS-CoV-2 antibody test kits distributed or developed in Japan

**DOI:** 10.4155/bio-2021-0254

**Published:** 2022-03-02

**Authors:** Hiroko Shibata, Kazuko Nishimura, Takuya Maeda, Masamitsu Honma, Yukihiro Goda, Akiko Ishii-Watabe, Yoshiro Saito

**Affiliations:** ^1^Division of Biological Chemistry & Biologicals, National Institute of Health Sciences, Kanagawa, 210-9501, Japan; ^2^Department of Clinical Laboratory, Saitama Medical University Hospital, Saitama, 350-0495, Japan; ^3^National Institute of Health Sciences, Kanagawa, 210-9501, Japan; ^4^Division of Medicinal Safety Science, National Institute of Health Sciences, Kanagawa, 210-9501, Japan

**Keywords:** analytical performance, anti-SARS-CoV-2 antibody test, antibody titer, automated analyzer, cut-off titer, cut-off value, ELISA, immunochromatography

## Abstract

**Background:** With the spread of COVID-19, anti-SARS-CoV-2 antibody tests have been utilized. Herein we evaluated the analytical performance of anti-SARS-CoV-2 antibody test kits using a new reference standard prepared from COVID-19 patient sera. **Methods:** Fifty-seven kits in total (16 immunochromatography types, 11 ELISA types and 30 types for automated analyzers) were examined. By measuring serially diluted reference standards, the maximum dilution factor showing a positive result and its precision were investigated. **Results:** The measured cut-off titers varied largely depending on the antibody kit; however, the variability was small, with the titers obtained by each kit being within twofold in most cases. **Conclusion:** The current results suggest that a suitable kit should be selected depending on the intended purpose.

Severe acute respiratory syndrome coronavirus 2 (SARS-CoV-2), which causes COVID-19, has been prevalent worldwide since December 2019 [1]. The WHO Emergency Committee agreed that this outbreak met the criteria for a public health emergency of international concern on 30 January 2020, later characterizing COVID-19 as a pandemic on 11 March 2020. As of 7 October 2021, more than 235 million and 4.8 million people worldwide were confirmed to be infected and to have died, respectively, from this infectious disease [2]. COVID-19 remains a serious threat to global health and the global economy. SARS-CoV-2 is an RNA virus with a genome of approximately 30 kb that generates surface spike glycoprotein (S protein), nucleocapsid protein (N protein) and other envelope membranes and accessory proteins [3]. The S protein binds to its receptors, mainly ACE2, and subsequently invades human cells for replication.

To prevent the spread of SARS-CoV-2, accurate diagnosis of infection is a prerequisite; RT-PCR and antigen testing are currently used for definitive diagnosis. On the other hand, serological (antibody) tests, which typically detect IgG and IgM directed against SARS-CoV-2, are considered to be useful for detecting past infections and thus may reflect the rate of recent infection experiences in tested populations. In a meta-analysis, most adults infected with SARS-CoV-2 developed specific IgM and IgG antibodies which emerged at around 7–12 days and peaked at around 20 and 25 days after symptom onset [4]. A large-scale antibody survey has been performed for residents and at-risk populations, with positivity rates of 1.5–20% [5]. A UK study of 12,541 healthcare workers showed that 223 of 11,364 subjects (1.96%) who were negative for anti-S protein IgG at baseline developed positive PCR test results by around 31 weeks compared with only two of 1265 (0.16%) who were positive for anti-S protein IgG at baseline, suggesting that positivity for anti-SARS-CoV-2 IgG can be a marker for reduced reinfection risk [6]. A significant reduction in the risk for SARS-CoV-2 infection has been reported in seropositive participants compared with seronegative controls [7,8]. Thus, the reliability of testing results for antibodies to SARS-CoV-2 is important for planning administrative measures in national health policy-making against SARS-CoV-2, including immunity passports.

The US FDA started to grant emergency use authorization (EUA) for antibody test kits reaching certain levels of test reliability in May 2020 [9]; as of 7 October 2021, 88 products had obtained this EUA [10]. The most recent version (6 Oct 2021) [11] of the EUA submission form requires data on clinical agreement using at least 30 RT-PCR-positive individual samples and 75 unique RT-PCR-negative subject samples (or samples collected before December 2019), giving a minimum of 90% overall (IgG + IgM) positive percent agreement (PPA), or a minimum of 70% PPA for IgM only and 90% PPA for IgG only, and 95% overall negative percent agreement. The UK Medicines and Healthcare Products Regulatory Agency also issued guidance that was updated on 5 October 2021 [12]. For any point-of-care/near-patient test or self-test, its diagnostic sensitivity and specificity should be >98% (95% CI: 96–100%) using at least 200 each of confirmed positive and negative specimens (or specimens collected at least 6 months before the known appearance of the virus). These guidelines also require no known cross-reactivity with other coronaviruses and other common respiratory pathogens as desired characteristics. The US Centers for Disease Control and Prevention also publicized an interim general guideline for COVID-19 antibody testing [13].

SARS-CoV-2 antibody tests can be categorized by the antigens used in the kits, antibodies to be detected and assay principles. Recombinant S protein, N protein or both are used as antigens in various kits. The antibodies to be detected may be IgG, IgM or total immunoglobulin (Ig). The assay principle utilizes a separation method and/or a detection method. Immunochromatography is a simple and easy-to-handle system for separation and detection and can be used without any specific equipment. ELISA employs an enzyme-labeled detection reagent and is usually used in research laboratories. The kits for automated analyzers cover many kinds of assay principles, including chemiluminescence immunoassay, chemiluminescent microparticle immunoassay, enzyme-linked fluorescence immunoassay, electrochemiluminescence immunoassay and fluorescent microsphere immunoassay. Because the analytical performance of antibody test kits can vary greatly depending on the materials and methods used for the kits, the reliability of test results has become a concern [14].

US governmental laboratories have evaluated serological tests using either of the four panels on 30 SARS-CoV-2 antibody-positive samples and 80 negative samples and showed data on sensitivity, specificity and the positive and negative predictive values of various kits. As of 7 October 2021, 37 kits had received EUA, but 65 kits did not reach the level for authorization and should not be used [15]. Public Health England also tested ten representative kits (nine for automatic clinical laboratory systems and one for ELISA) regarding their sensitivity, specificity, positive and negative predictive values and precision [16]. Many published studies have also assessed the analytical performance of available antibody test kits using panels of seropositive and seronegative samples, with some of them comparing sensitivity and specificity values between different kits [17–24]. These are very valuable results when the reliability of an individual kit is assessed for governmental authorization and the selection of a research kit for specific purposes. However, the amounts and characteristics of SARS-CoV-2-specific IgG/IgM would vary depending on the samples used within each study; thus, if the used plasma/serum panels were different between the assays, the deduced values of sensitivity and specificity would also be different even if the same antibody kit were used. Therefore the diagnostic sensitivity and specificity values cannot be directly compared if the same panels are not used.

To compare kit performance, a method other than using panels of seropositive and seronegative samples is to utilize standardized plasma/serum. In the present study we prepared pooled serum from COVID-19 patients as a reference standard, then evaluated the maximum dilution factor giving positive results and its precision on 57 antibody test kits, which were commercially available or developed in Japan as of October 2020, adhering to a request from the Ministry of Health, Labor and Welfare of Japan.

## Materials & methods

### Serum samples from COVID-19 patients

Sera were collected at Saitama Medical University Hospital and Nagasaki University from COVID-19 patients who were confirmed to have SARS-CoV-2 infection by quantitative RT-PCR or reverse-transcription loop-mediated isothermal amplification (Loopamp^®^ 2019-SARS-CoV-2 Detection Reagent Kit; Eiken Chemical, Tokyo, Japan), which were approved for *in vitro* diagnostics. Serum collection and subsequent analysis were performed with the approval of the ethics committees at the National Institute of Health Sciences (NIHS), Saitama Medical University Hospital, and Nagasaki University. Written informed consent or opt-out consent was obtained from all participants. Serum was prepared via standard methods using blood collected from an arm vein.

### Preparation of reference standard

Thirty-four serum samples from COVID-19 patients were mixed gently on ice to prepare as the reference standard (referred to as NIHS-RS). The pooled serum was dispensed in aliquots and stored at -80°C until being distributed to the laboratories. The antibody titer of NIHS-RS was evaluated using anti-SARS-CoV-2 antibody test kits for SARS-CoV-2 ELISA (IgG), SARS-CoV-2 NCP ELISA (IgG) and SARS-CoV-2 NCP ELISA (IgM) (Euroimmun AG, Lübeck, Germany), and characterized by comparison with the WHO international standard (WHO-IS, NIBSC code: 20/136) for anti-SARS-CoV-2 immunoglobulin.

### Preparation of serum for dilution

The pooled sera for dilution (hereinafter referred to as the ‘serum for dilution’), which were used for analyte preparation and were also used as a negative control sample, were prepared by mixing the following human pooled serum on ice: 40 ml of SER019A020G002 (collected in May 2019) and 150 ml of SER019A050G005 (collected in March 2020), both purchased from Biopredic International (Rennes, France), and 50 ml of CT-0375491-050 (collected in January 2019), purchased from Clinical Trials Laboratory Services Ltd (London, UK). The anti-S and anti-N antibodies in the pooled serum were confirmed to be negative by several ELISA and immunochromatographic kits. The serum for dilution was dispensed in aliquots and stored at -80°C until being distributed to the laboratories.

### Anti-SARS-CoV-2 antibody kits tested

In this study, 30 types of kits for automated analyzers, 11 types of ELISA kits and 16 types of immunochromatography kits were used (Table 1 & Supplementary Table 1). They comprised a variety of antibody test kits available in Japan in October 2020. Anti-SARS-CoV-2 antibodies were measured with S protein, N protein or both S and N proteins as antigens, depending on the kit. In the case of S protein, there were kits using S protein domain 1 and kits using the receptor binding domain. Although details were not disclosed regarding the expression system (mammalian cells or non-mammalian cells, such as *Escherichia coli*, or cell-free protein synthesis) of antigen proteins used, the expression systems seemed to differ among kits. Analytes for the kits used were IgG, IgM or total immunoglobulin.

**Table 1.** List of antibody test kits evaluated and participants in this study.

(A) Kits for automated analyzer		
Participants of the study	ID	Antibody test
Abbott Japan LLC	A-1-1	ARCHITECT SARS-CoV-2 IgG II Quant
	A-1-2	Alinity SARS-CoV-2 IgG
	A-1-3	Alinity SARS-CoV-2 IgM
Research Institute of Tuberculosis	A-13-1	Abbott ARCHITECT SARS-CoV-2 IgG
	A-13-2	Abbott ARCHITECT SARS-CoV-2 IgM
Medical and Biological Laboratories Co., Ltd.	A-2-1	iFlash-SARS-CoV-2 IgG
	A-2-2	iFlash-SARS-CoV-2 IgM
Ortho Clinical Diagnostics Inc.	A-3-1	VITROS Immunodiagnostic Products Anti-SARS-CoV-2 IgG Reagent Pack
	A-3-2	VITROS Immunodiagnostic Products Anti-SARS-CoV-2 Total Reagent Pack
Thermo Fisher Diagnostics K.K	A-4	EliA SARS-CoV-2-Sp1 IgG
Sysmex Co.	A-5-1	(RUO) HISCL SARS-CoV-2 S-IgG
	A-5-2	(RUO) HISCL SARS-CoV-2 S-IgM
	A-5-3	(RUO) HISCL SARS-CoV-2 N-IgG
	A-5-4	(RUO) HISCL SARS-CoV-2 N-IgM
Siemens Healthcare Diagnostics Inc.	A-6-1	Atellica IM SARS CoV-2 IgG (COV2G)
	A-6-2	ADVIA Centaur SARS-CoV-2 IgG (COV2G)
	A-6-3	Dimension EXL SARS-CoV-2 IgG LOCI (Dimension EXL SARS-CoV-2 IgG (CV2G))
	A-6-4	Atellica IM SARS-CoV-2 Total (COV2T)
	A-6-5	Dimension EXL SARS-CoV-2 Total LOCI (Dimension EXL SARS-CoV-2 Total antibody assay (CV2T))
Tosoh Corporation	A-7-1	AIA-CL SARS-CoV-2 NP-IgG
	A-7-2	AIA-CL SARS-CoV-2 NP-Total
	A-7-3	AIA-CL SARS-CoV-2 SP-IgG
	A-7-4	AIA-CL SARS-CoV-2 SP-Total
Bio-Rad Laboratories, Inc.	A-8	BioPlex SARS-CoV-2 IgG Panel
FUJIFILM Wako Pure Chemical Corporation	A-9	Accuraseed COVID-19 antibody (Prototype)
FUJIREBIO Inc.	A-10	SARS-CoV-2 Antibody test (Prototype)
Beckman Coulter, Inc.	A-11-1	Access SARS-CoV-2 IgG Reagent Kit
	A-11-2	Access SARS-CoV-2 IgM Reagent Kit
Roche Diagnostics K.K.	A-12-1	Elecsys Anti-SARS-CoV-2 RUO
	A-12-2	Elecsys Anti-SARS-CoV-2 S RUO

(B) ELISA kits		
Participants of the study	ID	Antibody test
Kanto Chemical Co., Inc.	E-1	Cica EliTo SARS-CoV-2 IgG ELISA Kit (Prototype)
Cellspect Co., Ltd,	E-2-1	QuaResearch COVID-19 Human IgM IgG ELISA Kit (Spike Protein)
	E-2-2	QuaResearch COVID-19 Human IgM IgG ELISA Kit (Nucleocapsid Protein)
Denka Co., Ltd.	E-3-1	COVID-19 S-IgG (in Development)
	E-3-2	COVID-19 RBD-IgG (in Development)
	E-3-3	COVID-19 N-IgG (in Development)
FUJIFILM Wako Pure Chemical Corporation	E-4	COVID-19 IgG ELISA Kit Wako (S-RBD)
EUROIMMUN Japan Co., Ltd.	E-5-1	Anti-SARS-CoV-2 ELISA (IgG)
	E-5-2	Anti-SARS-CoV-2 NCP ELISA (IgG)
	E-5-3	Anti-SARS-CoV-2 NCP ELISA(IgM)
Bio-Rad Laboratories, Inc.	E-6	Platelia SARS-CoV-2 Total Ab

(C) Immunochromatography kits		
Participants of the study	ID	Antibody test
LSI Medience Co.	I-1	PRORAST SARS-CoV-2 IgM/IgG
KAINOS Laboratories, Inc.	I-2	WANTAI SARS-CoV-2 Total Ab Rapid Test
Kanto Chemical Co., Inc.	I-3	Cica Immunotest SARS-CoV-2 IgG EX
Kyokuto Pharmaceutical Industrial Co., Ltd.	I-4	Autobio Anti-SARS-CoV-2 Rapid Test
Quantum Molecular Diagnostics LLC	I-5	SARS-CoV-2 IgM & IgG Quantum Dot Immunoassay (RUO)
Kurabo Industries Ltd.	I-6	SARS-CoV-2 Antibody Detection Kit (IgM/IgG) (Immunochromatograph method)
Kohjin Bio Co., Ltd.	I-7	KBM COVID-19 IgG/IgM
Sanwa Kagaku Kenkyusyo Co., Ltd.	I-8	Wondfo SARS-CoV-2 Antibody Test (Lateral Flow Method)
SHIONOGI & Co., Ltd.	I-9	2019-nCoV IgG/IgM Detection Kit (Colloidal Gold-Based)
SHIMA Laboratories Co., Ltd.	I-10	LYHER Novel Coronavirus (2019-nCoV) IgM/IgG Antibody Combo Test Kit (Colloidal Gold)
Nichirei Biosciences Inc.	I-11	STANDARD Q COVID-19 IgM/IgG Duo Test
Nissui Pharmaceutical Co., Ltd.	I-12	NG-Test IgG-IgM COVID-19
Roche Diagnostics K.K.	I-13	SARS-CoV-2 Rapid Antibody Test (RUO)
Cellspect Co., Ltd,	I-14	QuaResearch COVID-19 IgG LF
National Institute of Health Sciences	I-15-1	Innovita 2019-nCoV Ab Test (Colloidal Gold)
	I-15-2	Cellex qSARS-CoV-2 IgG/IgM Cassette Rapid Test

Ab: Antibody; RBD: Receptor binding domain.

Regarding the kits for automated analyzers, all kits used their own calibrator reagents to determine the positivity/negativity of the anti-SARS-CoV-2 antibody for the samples, and almost all data displayed by the analyzers were calculated values as an index or unit based on the response value of the calibrator. Each kit has its own calculation method, and the rationales were not disclosed. The most frequently used assay principle was the chemiluminescence immunoassay. A two-step assay format was used in most kits to detect IgG or IgM, whereas the bridging format (where antigens labeled by two different tags are used) was used for total Ig detection.

Most of the ELISA kits also used their own control reagents to determine the positivity/negativity of the antibody. In the protocol of each kit, the recommended dilution factor of samples was approximately 10, 100, 200 or 200 to 2000. All ELISA kits adopted a two-step assay using an antigen-precoated plate and peroxidase-labeled anti-human IgG or IgM antibodies.

Many immunochromatography kits can detect both IgG and IgM on the same cassette or strip. The sample volume required for the test was 10–20 μl. The major assay principle of the immunochromatography kits was that colloidal gold coated with SARS-CoV-2 antigens was mounted on the end of the strip, and anti-human IgG or IgM were immobilized on the test zone. Inversely, there were kits in which SARS-CoV-2 antigens were immobilized on the test zone. One kit (I-5) included an analyzer that quantifies the fluorescence intensity of the test and control lines. The I-15 kits were purchased and examined by NIHS as the kits that obtained an EUA from the FDA at that time.

### Measurement using each anti-SARS-CoV-2 antibody kit

**Figure 1.**
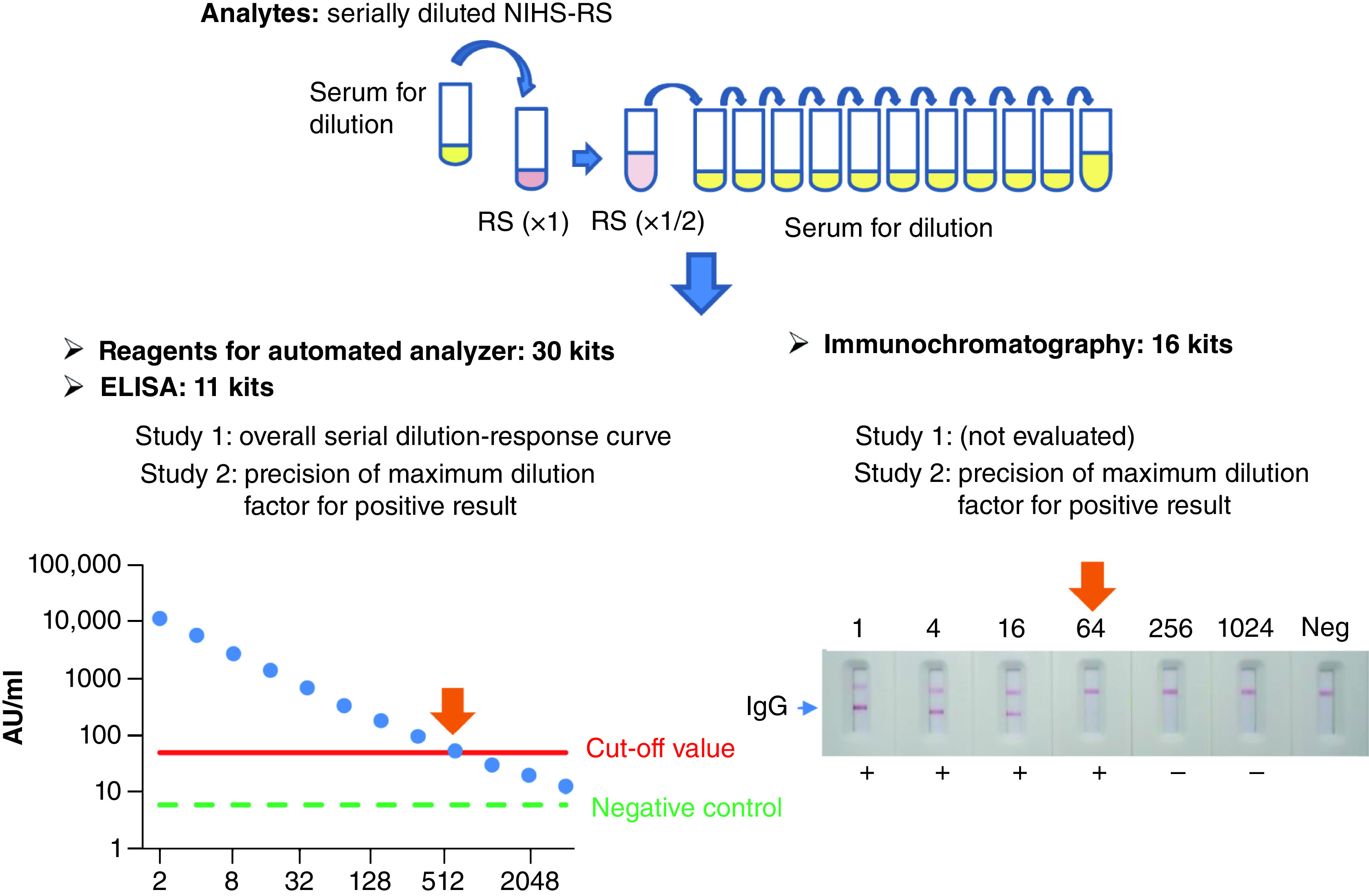
Outline of the study design. The analytes were prepared by serial dilution of reference standard independently for each run. In study 1, the analytes were tested using kits for an automated analyzer and ELISA, and the dilution factor corresponding to the cut-off titer was calculated by linear regression from the calibration curve. In study 2, the maximum dilution factor resulting in a positive result was obtained for all kits of the automated analyzer, ELISA and immunochromatography in three runs, and the precision was evaluated. NIHS-RS: National Institute of Health Sciences Reference Standard.

The outline of our study is shown in Figure 1. The anti-SARS-CoV-2 antibody kits are listed in Table 1, and the measurements were performed in each organization (manufacturer or distributor) or contracted laboratory in this study. Additional information on each antibody test kit is summarized in Supplementary Table 1. NIHS-RS was serially twofold diluted with the serum for dilution and measured according to the instructions of each kit in the laboratory of each organization involved in this study. Positive/negative determination was also conducted by each organization according to the instructions of each kit. Two studies were conducted: study 1 was to obtain the entire serial dilution–response curve of kits for automated analyzer and ELISA, while study 2 was to evaluate the precision of the cut-off titers of all kits.

#### Study 1

To obtain the entire serial dilution–response curve for the automated analyzer and ELISA kits, the analytes were prepared by twofold serial dilution of NIHS-RS across 12 steps, then the samples were assayed in triplicate. The dilution factor that intersects a cut-off value (i.e., the cut-off titer) was estimated from a linear regression using the two points on both sides of a cut-off value on the serial dilution–response curve.

#### Study 2

To evaluate the precision of the maximum dilution factor corresponding to a positive result, the analytes were prepared by a twofold serial dilution of NIHS-RS. For the automated analyzer and ELISA kits, the range of serial dilutions was set to 16 and eight steps, respectively. For the immunochromatography kit, the range of serial dilutions was determined during the experiments in which the test results were negative. The samples were assayed in triplicate for automated analyzer and ELISA kits, or in duplicate for immunochromatography kits, and the assay was conducted three times on different days. The maximum dilution factor for a positive result (i.e., the cut-off titer) was evaluated for each measurement.

## Results

### Characterization of NIHS-RS used in this study

**Figure 2.**
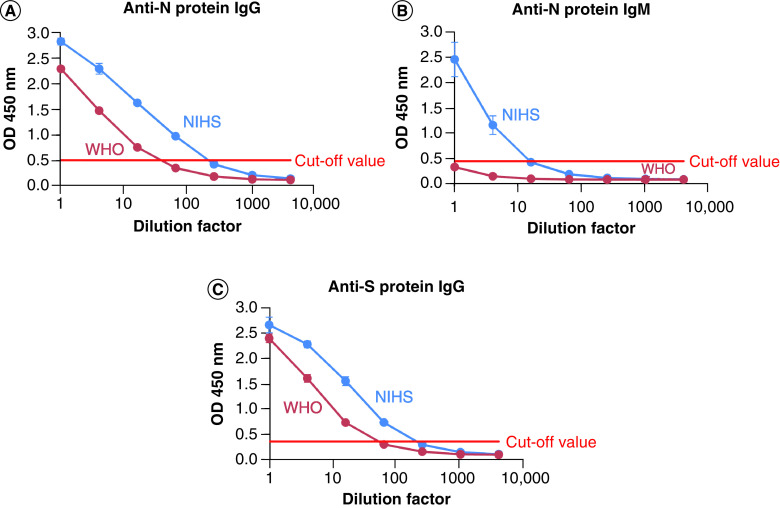
Characterization of reference standards used in this study. The antibody response of NIHS-RS and WHO-IS 20/136 was compared using the Euroimmun ELISA kit. **(A)** Anti-N protein IgG. **(B)** Anti-N protein IgM. **(C)** Anti-S protein IgG. NIHS-RS:NIHS: National Institute of Health Sciences Reference Standard; WHO: WHO International Standard (Code no. 20/136).

The antibody titers of NIHS-RS and WHO-IS were measured using three ELISA kits with high clinical sensitivity and specificity which are commonly used worldwide (Figure 2). Each ELISA kit can detect IgG against SARS-CoV-2 S protein, IgG against N protein, or IgM against N protein in serum samples. The responses for NIHS-RS were higher than those of WHO-IS for all kits. In particular, the response of IgM in NIHS-RS to N protein was much higher than that in WHO-IS. The antibody levels of IgG against N and S proteins in NIHS-RS were calculated to be 6.117- and 3.912-times higher than in WHO-IS at the cut-off values in the ELISA. Because the antibody level of WHO-IS is assigned as 1000 binding antibody units/ml (BAU/ml), the antibody titer of NIHS-RS was estimated to be approximately 6000 and 4000 BAU/ml for IgG against N and S proteins, respectively. It is noteworthy that NIHS-RS prepared from 34 serum samples had a sufficient variety of anti-SARS-CoV-2 antibodies and thus representativeness compared with the WHO-IS (20/136) prepared from 11 plasma samples. These data indicated that, in terms of antibody content and diversity, the NIHS-RS was suitable for use in the performance evaluation of anti-SARS-CoV-2 antibody test kits in the collaborative study.

### Study 1: Evaluation of cut-off titer of kits for automated analyzer & ELISA

**Figure 3.**
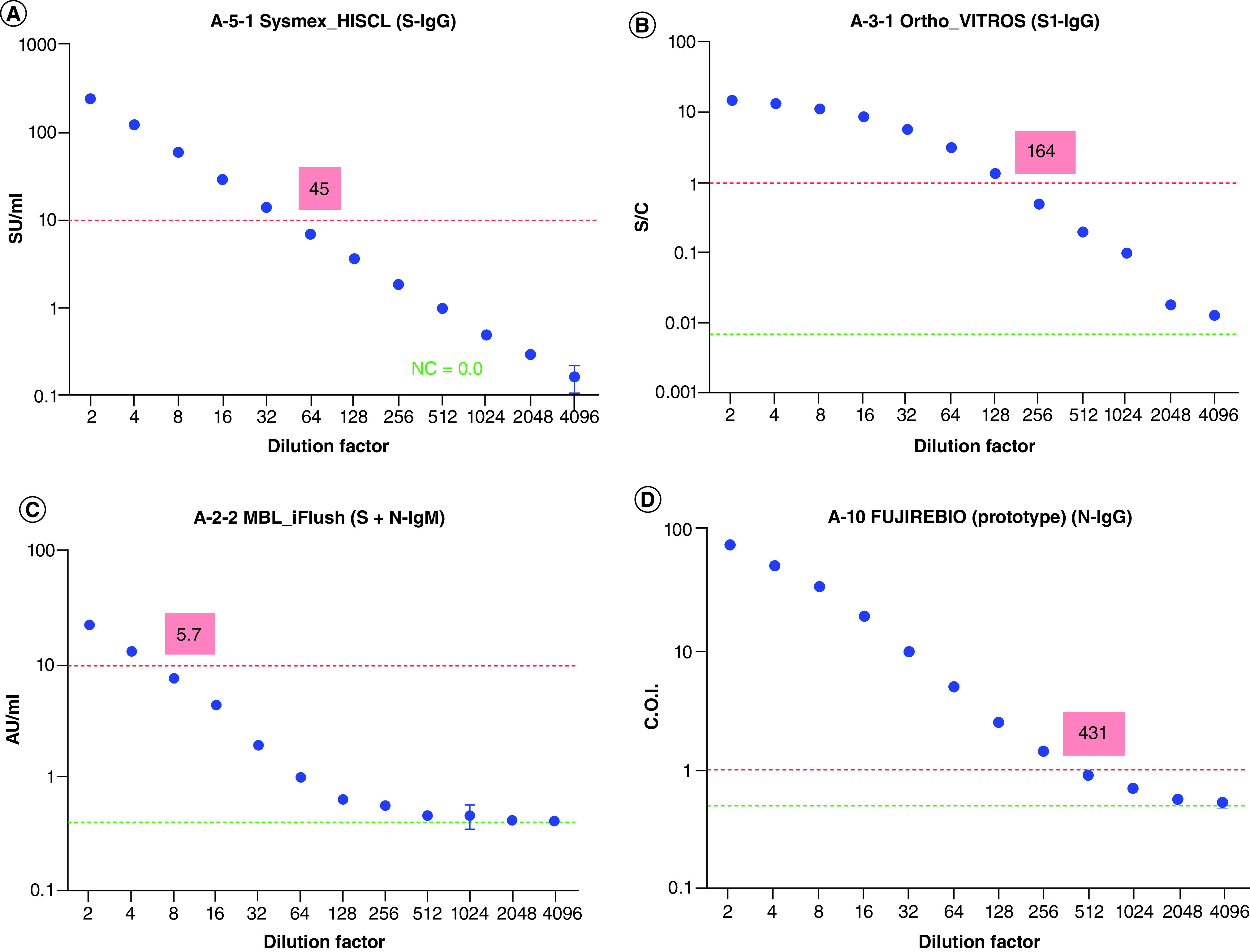
Representative serial dilution–response curves obtained by kits which generate numerical responses. Serially diluted reference standard (NIHS-RS) was analyzed using each kit, and the response was plotted against the dilution factor. Four typical types of serial dilution–response curves obtained using kits for automated analyzers are shown. Some kits showed **(A)** a linear response or **(B)** a non-linear response. In some kits, the cut-off value was **(C)** set well above NC or **(D)** set near NC. Red dotted line: cut-off value, green dotted line: response of negative control, numerical number in red box: cut-off titer. NC: Negative control (NC = 0 means that the response of NC was defined as 0); NIHS-RS: National Institute of Health Sciences Reference Standard.

In study 1, the serial dilution–response curve and cut-off titer were examined using kits for automated analyzer and ELISA. The serial dilution–response curves obtained for all kits examined, and the cut-off values, as well as the measured values of the negative control (serum for dilution) of each kit are graphically presented in Supplementary Figure 1. The dilution factor that intersected the cut-off value (i.e., the cut-off titer) is shown in each graph and these are listed in Tables 2 & 3. The cut-off titer depended on the kit. Typical serial dilution–response curves are shown in Figure 3. All values of the negative control were sufficiently low, and there were no issues regarding the level of the background for all kits. The shape of the serial dilution–response curve varied among kits: some were straight (Figure 3A) and some were curved because of saturation of response (Figure 3B). A decrease in responses with an increase in dilution factor was observed up to the maximum dilution factor, except for some antibody kits. Some kits showing steep slopes around the cut-off value had cut-off values well above those of the negative control (Figure 3C); in contrast, the cut-off values with a lower point of the curves were near the values of the negative control in some kits (Figure 3D).

**Table 2.** Cut-off titer evaluation using serially diluted reference serum: Kits for automated analyzer.

ID	Antibody test	Antigen	Analyte Ab	Study 1	Study 2											
				Cut-off titer^†^	Cut-off titer^‡^	Precision: ratio of positive results in 3 runs of triplicate assay										
						×2	×4	×8	×16	×32	×64	×128	×256	×512	×1024	×2048
A-1-1	ARCHITECT SARS-CoV-2 IgG II Quant	S1-RBD	IgG	553	256	-	-	-	9/9	9/9	9/9	9/9	9/9	6/9	0/9	0/9
A-1-2	Alinity SARS-CoV-2 IgG	N	IgG	142	64	-	-	-	9/9	9/9	9/9	4/9	0/9	0/9	0/9	0/9
A-1-3	Alinity SARS-CoV-2 IgM	S	IgM	57	32	-	-	-	9/9	9/9	0/9	0/9	0/9	0/9	-	-
A-13-1	Abbott ARCHITECT SARS-CoV-2 IgG	N	IgG	118	64	-	-	-	9/9	9/9	9/9	0/9	0/9	0/9	0/9	0/9
A-13-2	Abbott ARCHITECT SARS-CoV-2 IgM	S	IgM	50	32	-	-	-	9/9	9/9	0/9	0/9	0/9	0/9	-	-
A-2-1	iFlash-SARS-CoV-2 IgG	S + N	IgG	179	128	-	-	-	9/9	9/9	9/9	9/9	0/9	0/9	0/9	0/9
A-2-2	iFlash-SARS-CoV-2 IgM	S + N	IgM	6	4	9/9	9/9	0/9	0/9	0/9	0/9	-	-	-	-	-
A-3-1	VITROS Anti-SARS-CoV-2 IgG Reagent Pack	S1	IgG	164	128	-	-	-	9/9	9/9	9/9	9/9	0/9	0/9	0/9	0/9
A-3-2	VITROS Anti-SARS-CoV-2 Total Reagent Pack	S1	Total Ig	1042	512	-	-	-	9/9	9/9	9/9	9/9	9/9	9/9	5/9	0/9
A-4	EliA SARS-CoV-2-Sp1 IgG	S1	IgG	81	32	-	-	-	9/9	9/9	7/9	0/9	0/9	0/9	0/9	0/9
A-5-1	(RUO)HISCL SARS-CoV-2 S-IgG	S	IgG	45	32	-	-	-	9/9	9/9	0/9	0/9	0/9	0/9	0/9	0/9
A-5-2	(RUO)HISCL SARS-CoV-2 S-IgM	S	IgM	44	32	-	-	-	9/9	9/9	0/9	0/9	0/9	0/9	0/9	0/9
A-5-3	(RUO)HISCL SARS-CoV-2 N-IgG	N	IgG	115	64	-	-	-	9/9	9/9	9/9	0/9	0/9	0/9	0/9	0/9
A-5-4	(RUO)HISCL SARS-CoV-2 N-IgM	N	IgM	9	8	-	9/9	9/9	0/9	0/9	0/9	0/9	-	-	-	-
A-6-1	Atellica IM SARS CoV-2 IgG (COV2G)	S1-RBD	IgG	171	128	-	-	-	9/9	9/9	9/9	9/9	0/9	0/9	0/9	0/9
A-6-2	ADVIA Centaur SARS-CoV-2 IgG (COV2G)	S1-RBD	IgG	156	128	-	-	-	9/9	9/9	9/9	9/9	0/9	0/9	0/9	0/9
A-6-3	Dimension EXL SARS-CoV-2 IgG LOCI	S1-RBD	IgG	129	128	-	-	-	9/9	9/9	9/9	9/9	0/9	0/9	0/9	0/9
A-6-4	Atellica IM SARS-CoV-2 Total (COV2T)	S1-RBD	Total Ig	162	128	-	-	-	9/9	9/9	9/9	9/9	0/9	0/9	0/9	0/9
A-6-5	Dimension EXL SARS-CoV-2 Total LOCI	S1-RBD	Total Ig	112	64	-	-	-	9/9	9/9	9/9	8/9	0/9	0/9	0/9	0/9
A-7-1	AIA-CL SARS-CoV-2 NP-IgG	N	IgG	114	64	-	-	-	9/9	9/9	9/9	0/9	0/9	0/9	0/9	0/9
A-7-2	AIA-CL SARS-CoV-2 NP-Tota	N	Total Ig	78	64	-	-	-	9/9	9/9	9/9	0/9	0/9	0/9	0/9	0/9
A-7-3	AIA-CL SARS-CoV-2 SP-IgG	S	IgG	113	64	-	-	-	9/9	9/9	9/9	0/9	0/9	0/9	0/9	0/9
A-7-4	AIA-CL SARS-CoV-2 SP-Total	S	Total Ig	1531	1024	-	-	-	9/9	9/9	9/9	9/9	9/9	9/9	9/9	0/9
A-8-1	BioPlex SARS-CoV-2 IgG Panel	S1	IgG	128	64	-	-	-	9/9	9/9	9/9	3/9	0/9	0/9	0/9	0/9
A-8-2		S2	IgG	20	16	-	-	-	9/9	0/9	0/9	0/9	0/9	0/9	0/9	0/9
A-8-3		S-RBD	IgG	213	128	-	-	-	9/9	9/9	9/9	9/9	0/9	0/9	0/9	0/9
A-8-4		N	IgG	84	64	-	-	-	9/9	9/9	9/9	0/9	0/9	0/9	0/9	0/9
A-9	Accuraseed COVID-19 antibody (Prototype)	S-RBD	IgG	143	128	-	-	-	9/9	9/9	9/9	9/9	0/9	0/9	0/9	0/9
A-10	SARS-CoV-2 Antibody test (Prototype)	N	IgG	431	256	-	-	-	9/9	9/9	9/9	9/9	9/9	3/9	0/9	0/9
A-11-1	Access SARS-CoV-2 IgG Reagent Kit	S-RBD	IgG	105	64	-	-	-	9/9	9/9	9/9	0/9	0/9	0/9	0/9	0/9
A-11-2	Access SARS-CoV-2 IgM Reagent Kit	S-RBD	IgM	45	32	-	-	-	9/9	9/9	0/9	0/9	0/9	0/9	-	-
A-12-1	Elecsys Anti-SARS-CoV-2 RUO	N	Total Ig	116	64	-	-	-	9/9	9/9	9/9	1/9	0/9	0/9	0/9	0/9
A-12-2	Elecsys Anti-SARS-CoV-2 S RUO	S-RBD	Total Ig	511	512	-	-	-	9/9	9/9	9/9	9/9	9/9	9/9	0/9	0/9

† Calculated as a dilution factor that intersects the cut-off value on the serial dilution-response curve.

‡ Expressed as a maximum dilution factor that shows positive result for all measurements.

Ab: Antibody; Ig: Immunoglobulin; N: Nucleocapsid; RBD: Receptor binding domain; S: Spike.

**Table 3.** Cut-off titer evaluation using serially diluted reference serum: ELISA kits.

ID	Antibody test	Antigen	Analyte Ab	Study 1	Study 2											
				Cut-off titer^†^	Cut-off titer^‡^	Precision: ratio of positive results in 3 runs of triplicate assay										
						×2	×4	×8	×16	×32	×64	×128	×256	×512	×1024	×2048
E-1	Cica EliTo SARS-CoV-2 IgG ELISA Kit (Sample)	N	IgG	58	32	9/9	9/9	9/9	9/9	9/9	8/9	0/9	0/9	0/9	0/9	0/9
E-2-1-1 E-2-1-2	QuaResearch COVID-19 Human IgM IgG ELISA Kit (Spike Protein)	S	IgG	9	4	9/9	9/9	0/9	0/9	0/9	0/9	0/9	0/9	0/9	0/9	0/9
		S	IgM	< 2	< 2	0/9	0/9	0/9	0/9	0/9	0/9	0/9	0/9	0/9	0/9	0/9
E-2-2-1 E-2-2-2	QuaResearch COVID-19 Human IgM IgG ELISA Kit (Nucleocapsid Protein)	N	IgG	65	32	9/9	9/9	9/9	9/9	9/9	8/9	0/9	0/9	0/9	0/9	0/9
		N	IgM	3	2	9/9	3/9	0/9	0/9	0/9	0/9	0/9	0/9	0/9	0/9	0/9
E-3-1	COVID-19 S-IgG (in Development)	S	IgG	89	64	9/9	9/9	9/9	9/9	9/9	9/9	0/9	0/9	0/9	-	-
E-3-2	COVID-19 RBD-IgG (in Development)	S-RBD	IgG	72	32	9/9	9/9	9/9	9/9	9/9	6/9	0/9	0/9	0/9	-	-
E-3-3	COVID-19 N-IgG (in Development)	N	IgG	96	64	9/9	9/9	9/9	9/9	9/9	9/9	0/9	0/9	0/9	-	-
E-4	COVID-19 IgG ELISA Kit Wako (S-RBD)	S-RBD	IgG	166	128	9/9	9/9	9/9	9/9	9/9	9/9	9/9	0/9	0/9	-	-
E-5-1	Anti-SARS-CoV-2 ELISA (IgG)	S1	IgG	-	64	8/8	8/8	8/8	8/8	8/8	8/8	2/8	0/8	-	-	-
E-5-2	Anti-SARS-CoV-2 NCP ELISA (IgG)	N	IgG	20	8	9/9	9/9	9/9	7/9	6/9	6/9	0/9	0/9	-	-	-
E-5-3	Anti-SARS-CoV-2 NCP ELISA(IgM)	N	IgM	10	8	9/9	9/9	9/9	3/9	0/9	0/9	0/9	0/9	-	-	-
E-6	Platelia SARS-CoV-2 Total Ab	N	Total Ig	229	128	-	-	-	9/9	9/9	9/9	9/9	0/9	0/9	0/9	0/9

† Calculated as a dilution factor that intersects the cut-off value on the serial dilution-response curve.

‡ Expressed as a maximum dilution factor that shows positive result for all measurements.

Ab: Antibody; Ig: Immunoglobulin; N: Nucleocapsid; RBD: Receptor binding domain; S: Spike.

### Study 2: Evaluation of cut-off titer & its precision for all kits

**Figure 4.**
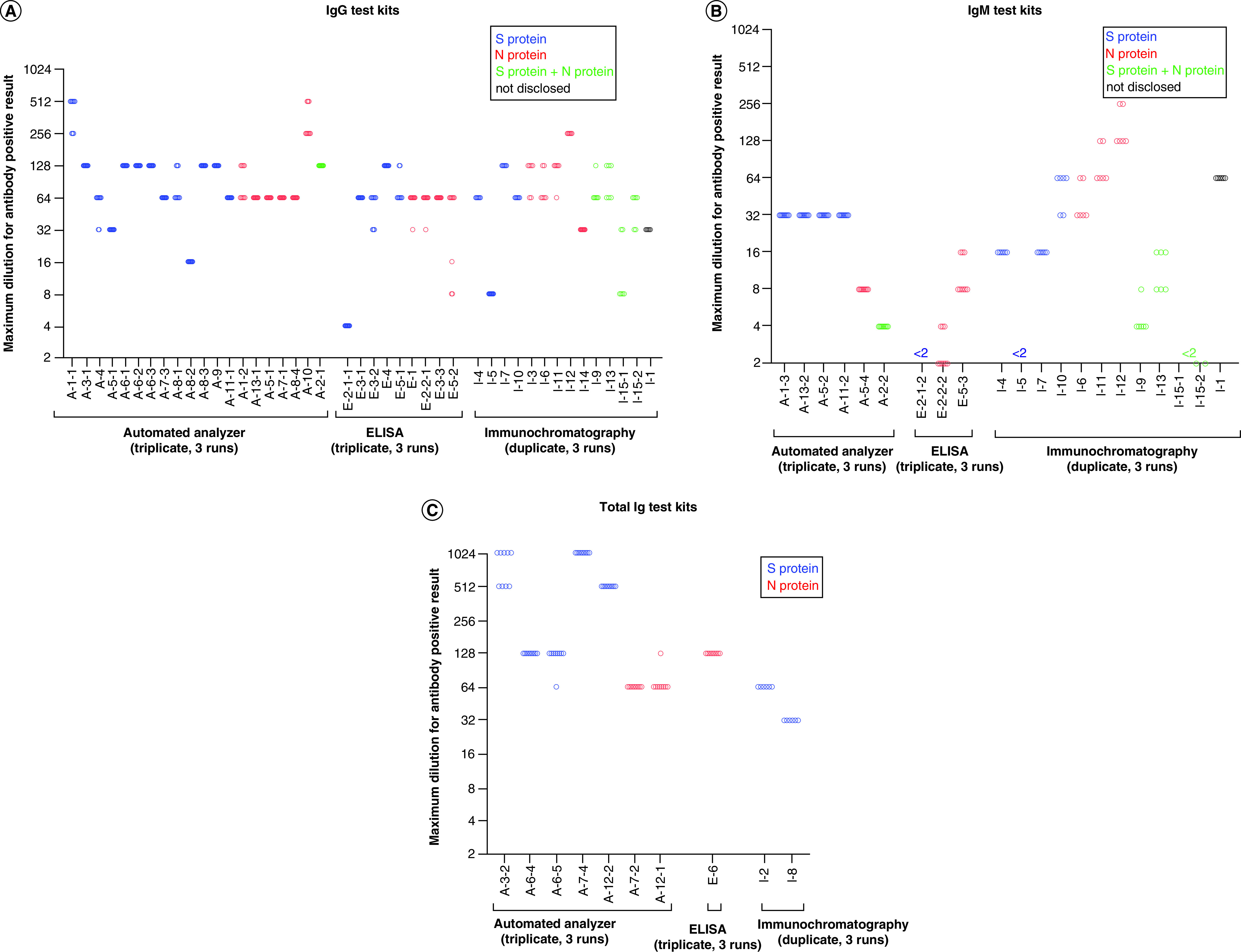
Precision of cut-off titer. Cut-off titers (n = 9 or 6) evaluated by triplicate or duplicate measurements of three runs were plotted. **(A)** IgG test kits. **(B)** IgM test kits. **(C)** Total immunoglobulin test kits.

In study 2, precision at the dilution factors just above the cut-off values was examined using kits for the automated analyzer, ELISA and immunochromatography. Because most immunochromatography kits do not generate numerical responses, it was impossible to determine the cut-off titer from the serial dilution–response curve of NIHS-RS. To compare the cut-off titer and its precision in all kits, including immunochromatography, serially diluted samples were measured three times in triplicate (3 × 3) or three times with duplicate (3 × 2), and then the maximum dilution factors that yielded positive results (cut-off titer) at all measurements (n = 9 or 6) in addition to the ratio of positive results at each dilution factor were evaluated. The detailed results of the cut-off titer and its precision evaluation using serially diluted NIHS-RS are summarized in Tables 2–4. The results are shown graphically in Figure 4 by classifying them into IgG, IgM and total Ig detection kits. The cut-off titer was from four- to 1024-fold in kits for automated analyzer, from more than two- to 128-fold in ELISA kits, and from more than two- to 256-fold in immunochromatography kits. Overall, the kits for the automated analyzer tended to show a higher cut-off titer, indicating that these kits can detect smaller amounts of antibodies against SARS-CoV-2 antigens.

**Table 4.** Cut-off titer evaluation using serially diluted reference serum: Immunochromatography kits.

ID	Antibody test	Antigen	Analyte Ab	Study 2											
				Cut-off titer^†^	Precision: ratio of positive results in 3 runs of triplicate assay										
					×2	×4	×8	×16	×32	×64	×128	×256	×512	×1024	×2048
I-1	PRORAST SARS-CoV-2 IgM/IgG	Not disclosed	IgG	32	6/6	6/6	6/6	6/6	6/6	0/6	0/6	0/6	-	-	-
			IgM	64	6/6	6/6	6/6	6/6	6/6	6/6	0/6	0/6	-	-	-
I-2	WANTAI SARS-CoV-2 Total Ab Rapid Test	S	Total Ig	64	2/2	2/2	2/2	6/6	6/6	6/6	0/6	0/6	-	-	-
I-3	Cica Immunotest SARS-CoV-2 IgG EX	N	IgG	64	6/6	6/6	6/6	6/6	6/6	6/6	4/6	0/6	-	-	-
I-4	Autobio Anti-SARS-CoV-2 Rapid Test	S	IgG	64	6/6	6/6	6/6	6/6	6/6	6/6	0/6	-	-	-	-
			IgM	16	6/6	6/6	6/6	6/6	0/6	0/6	0/6	-	-	-	-
I-5	SARS-CoV-2 IgM & IgG Quantum Dot Immunoassay (RUO)	S(RBD)	IgG	8	9/9	9/9	9/9	0/9	0/9	-	-	-	-	-	-
			IgM	< 2	0/9	0/9	0/9	0/9	0/9	-	-	-	-	-	-
I-6	SARS-CoV-2 Antibody Detection Kit (IgM/IgG)	N	IgG	64	6/6	6/6	6/6	6/6	6/6	6/6	2/6	0/6	-	-	-
			IgM	32	6/6	6/6	6/6	6/6	6/6	2/6	0/6	0/6	-	-	-
I-7	KBM COVID-19 IgG/IgM	S	IgG	128	6/6	6/6	6/6	6/6	6/6	6/6	6/6	0/6	0/6	-	-
			IgM	16	6/6	6/6	6/6	6/6	0/6	0/6	0/6	0/6	0/6	-	-
I-8	Wondfo SARS-CoV-2 Antibody Test	S	Total Ig	32	6/6	6/6	6/6	6/6	6/6	0/6	-	-	-	-	-
I-9	2019-nCoV IgG/IgM Detection Kit	S + N	IgG	64	6/6	6/6	6/6	6/6	6/6	6/6	1/6	0/6	-	-	-
			IgM	4	6/6	6/6	1/6	0/6	0/6	0/6	0/6	0/6	-	-	-
I-10	LYHER Novel Coronavirus (2019-nCoV) IgM/IgG Antibody Combo Test Kit	S	IgG	64	6/6	6/6	6/6	6/6	6/6	6/6	0/6	0/6	-	-	-
			IgM	32	6/6	6/6	6/6	6/6	6/6	4/6	0/6	0/6	-	-	-
I-11	STANDARD Q COVID-19 IgM/IgG Duo Test	N	IgG	64	6/6	6/6	6/6	6/6	6/6	6/6	5/6	0/6	-	-	-
			IgM	64	6/6	6/6	6/6	6/6	6/6	6/6	2/6	0/6	-	-	-
I-12	NG-Test IgG-IgM COVID-19	N	IgG	256	2/2	2/2	2/2	2/2	6/6	6/6	6/6	6/6	0/6	-	-
			IgM	128	2/2	2/2	2/2	2/2	6/6	6/6	6/6	2/6	0/6	-	-
I-13	SARS-CoV-2 Rapid Antibody Test RUO	S + N	IgG	64	6/6	6/6	6/6	6/6	6/6	6/6	3/6	0/6	-	-	-
			IgM	8	6/6	6/6	6/6	3/6	0/6	0/6	0/6	0/6	-	-	-
I-14	QuaResearch COVID-19 IgG LF	N	IgG	32	9/9	9/9	9/9	9/9	9/9	0/9	0/9	0/9	-	-	-
I-15-1	Innovita 2019-nCoV Ab Test	S1 + N	IgG	8	6/6	6/6	6/6	2/6	2/6	0/2	-	-	-	-	-
			IgM	< 2	0/6	0/6	0/6	0/6	0/6	0/2	-	-	-	-	-
I-15-2	Cellex qSARS-CoV-2 IgG/IgM Cassette Rapid Test	S + N	IgG	32	6/6	6/6	6/6	6/6	6/6	4/6	0/6	-	-	-	-
			IgM	< 2	2/6	0/6	0/6	0/6	0/6	0/6	0/6	-	-	-	-

† Expressed as a maximum dilution factor that shows positive result for all measurements.

Ab: Antibody; Ig: Immunoglobulin; LF: Lateral flow; N: Nucleocapsid; RBD: Receptor binding domain; S: Spike.

There were no kits that showed large variability in their cut-off titers as determined by three runs of triplicate or duplicate assays, because the titers obtained by each kit were within twofold in most cases (Figure 4 & Tables 2–4). In Figure 4, the cut-off titer can be compared within the same category of antigens and antibodies. For the kits for IgG detection, the cut-off titers were convergently distributed to 64- or 128-fold, although some kits resulted in lower cut-off titers. Regarding the kits for IgM detection, one ELISA kit and two immunochromatography kits could not detect antibodies even in twofold-diluted NIHS-RS samples. Comparing kits for the automated analyzer developed with the same brand name by an identical company, the kits for total Ig detection showed higher analytical sensitivity than the kits for IgG and IgM. Photographs of the immunochromatography tests are shown in Supplementary Figure 2.

## Discussion

Antibody tests can have health value for monitoring antibody responses in the COVID-19 pandemic and clinical utility in the recent SARS-CoV-2 infection outbreak [13]. Because antibody tests have many variations in their assay principles and individual performance characteristics, it is important to use an appropriate kit that can meet the intended purpose. Several studies have compared the performance of antibody test kits [25–28]. In the USA, independent evaluation of antibody tests using panels that were prepared from SARS-CoV-2 antibody-positive patient samples is being conducted to evaluate the performance characteristics of antibody tests [15]. In this openFDA study, sensitivity and specificity were compared using the panels, and the measured results, as well as the expected positive and negative predictive values, were reported. Our study also compared the performance characteristics of antibody test kits but is unique in that the serial dilution–response curves were generated using serially diluted common NIHS-RS samples, and the cut-off titers yielding positive results among kits were compared, providing the interesting characteristics of each kit.

The analytical performance was optimized and validated during the development of these kits. However, the clinical samples used for these steps differ depending on the kit or company; therefore the values indicating performance, such as diagnostic sensitivity and specificity, cannot be directly compared. Because the intended purpose of the antibody test kits examined here was to test whether the sample is antibody positive or negative, the key issue is setting the cut-off value that determines the positivity or negativity of the clinical samples examined. Thus we evaluated the cut-off values of the antibody test kits in a collaborative study using pooled COVID-19 patient serum (NIHS-RS) as an analyte.

As shown in Figure 3 & Supplementary Table 1, the generated serial dilution–response curve of each antibody test kit showed unique characteristics in terms of setting the y-axis, shape of the serial dilution–response curve, cut-off value and the difference between response levels of cut-off and negative control. The variety of y-axis plots may reflect the variety of cut-off setting methods based on the response of the calibrator(s). The difference between the response levels of the cut-off and negative control may have increased as a result of maximizing both the sensitivity and specificity of each kit. The difference in the cut-off values of these kits (Figure 4 & Tables 2–4) shows that one clinical sample can be judged as positive or negative depending on the kit used. These data also clearly showed that the data obtained using different kits cannot be directly compared.

As the factors generated such characteristic differences, the following were considered:

- There was no common reference standard for SARS-CoV-2 antibodies during the development of the kits;
- There was no clinical target value of antibody titer for the SARS-CoV-2 antibody test;
- Used antigen structure, as well as immobilization methods, was different among the kits;
- Assay principles that detect antibody bound on the antigen were different among the kits;
- Variability of the SARS-CoV-2 antibody-positive and -negative samples used during method development and validation varied among the kits.

Recently, the antibody level associated with 80% vaccine efficacy (VE) against primary symptomatic COVID-19 was reported to be 264 BAU/ml of anti-S IgG in the randomized efficacy trial of the ChAdOx1 nCoV019 (ADZ1222) vaccine in the UK [29]. Because the anti-S IgG level of NIHS-RS was estimated to be approximately 4000 BAU/ml, the antibody level of 80% VE corresponds to about 15-fold dilution of NIHS-RS. The antibody level associated with 50% VE was reported to be 29 BAU/ml, which corresponds to about 135-fold dilution of NIHS-RS anti-S IgG titer. The results of this study (Tables 2–4) indicate that most of the kits for anti-S IgG yielded a positive result for antibody titer of 80% VE; however, most of these kits yielded a negative result for the titer corresponding to 50% VE. Further investigation is necessary to clarify the required analytical sensitivity and cut-off titer for SARS-CoV-2 antibody test kits.

As shown in Figure 3 & Tables 3 & 4, there were three kits – E-2-1-2 (ELISA kit for anti-S IgM), I-5 (immunochromatography kit for anti-S IgM with a specific fluorescence reader) and I-15-1 (immunochromatography kit anti-S1 and N IgM) – in which IgM response was not detected. According to the manufacturers of these kits, the reason for this result is explained as follows:

- E-2-1-2: According to the instruction from the manufacturer, the intended use of this ELISA kit is to evaluate the relative response of antibodies in human samples but not to determine whether the sample is antibody positive or negative. The cut-off value used in this study (Figure 3) was the absolute value of UV absorption that was obtained during the method development, which gave a diagnostic sensitivity of 30–40% and a specificity of about 92% for IgM. This cut-off value can only be used in the laboratory of the manufacturer;
- I-5: In the label of the product, overall sensitivity and specificity are reported as 85.29 (58/61) and 97.44% (10/124), respectively [30]. In addition to the NIHS-RS, the IgM response was not detected when WHO-IS or WHO reference panel (NIBSC code no. 20/150) was used as the analyte. A possible reason for this discrepancy is the difference in antibody titer between the clinical samples used to evaluate the specificity of the kit and the analytes used in this study. The response of monoclonal IgM against SARS-CoV-2 S protein was confirmed by the manufacturer;
- I-15-1: In the label of the product, IgM sensitivity and specificity were reported as 93.3 (28/30) and 98.8% (79/80), respectively [31]. However, in addition to the NIHS-RS, the IgM response was not detected when WHO-IS or WHO reference panel was used as the analyte. A possible reason for this discrepancy is same as the case of kit I-5, or variability of analytical performance between different lots of the kit.

However, the vaccine efficacy against SARS-CoV-2 infection is dependent on the anti-SARS-CoV-2 IgG level, but not that of IgM. In this context, test sensitivity for anti-SARS-CoV-2 IgM antibody seems less significant clinically.

As shown in Figure 2, the NIHS-RS exhibited much higher titers of anti-N protein IgM than the WHO-IS when evaluated by ELISA. Our results indicate that the NIHS-RS was more suitable than WHO-IS (code no. 20/136) for evaluating the analytical performance of anti-N protein IgM kits. If WHO-IS was used, cut-off titers for IgM were more difficult to obtain. Meanwhile, it is also suggested that the analytical sensitivity of most IgM kits would not be sufficient for evaluating actual clinical samples.

In the USA, SARS-CoV-2 antibody tests are categorized as *in vitro* diagnostic tests, and an EUA has been granted to more than 80 products. On the other hand, antibody testing is not yet categorized as an *in vitro* diagnostic test in Japan; therefore there is no regulatory process, even for emergency use. This study has an impact as the largest study to reveal the characteristics of antibody tests available in Japan. However, the following points can be mentioned as limitations of this study. Firstly, bias in the test results depending on the analytical laboratory cannot be excluded because each kit was examined by its own manufacturer or distributer. Secondly, the NIHS-RS was prepared from patient serum collected before September 2020; therefore it is not clear whether the results obtained here can be applied to sera from patients who are infected with newer SARS-CoV-2 variants. Because antibody tests will be more important to evaluate the immune status of individual persons or persons in specific areas, the results of which are used to judge the necessity of vaccination and/or to establish the policy of regulation on COVID-19, it is critical to ensure the reliability of antibody test results. For this purpose, quantitative analysis will be more important. Our results that revealed the variety of dilution–response curves associated with many kinds of kits will also help understanding of the characteristics of antibody test kits for SARS-CoV-2.

## Conclusion

To ensure the reliability of antibody tests, standardization of antibody titer evaluation, as well as the establishment of required analytical sensitivity and cut-off values to give positive results, is important. WHO-IS plays a critical role in the standardization of antibody titers, although the amount of WHO-IS is limited and not yet used thoroughly. With respect to the required analytical sensitivity and cut-off value, data to reveal the relationship between antibody titer and clinical impact (e.g., prevention of SARS-CoV-2 infection) should be accumulated in the near future. Neutralizing antibody activity is also important when considering infection prevention. Evaluating the performance characteristics of neutralizing antibody test kits is a particularly important future issue.

## Future perspective

Although the use of antibody tests for SARS-CoV-2 is limited to monitoring the COVID-19 pandemic and is not currently recommended to assess immunity generated by the SARS-CoV-2 vaccine [13], these tests will become more important in evaluating the efficacy of vaccines and their duration. To satisfy this purpose, standardization of antibody titer evaluation and the titer required to prevent SARS-CoV-2 infection should be established. The antibody test for measuring neutralization activity is more important for this purpose. The critical part of the standardization of antibody titer evaluation is using the WHO-IS for SARS-CoV-2 antibodies. However, the WHO-IS is pooled human plasma, and thus the amount is limited. Use of an internal second standard appropriately calibrated by the WHO-IS is necessary for the quality control of antibody tests in Japan.

**Executive summary**

**Background**

- Many antibody test kits for SARS-CoV-2 have been developed and distributed; however, their analytical performance had not been evaluated and compared.

**Experimental**

- Analytical performance (i.e., cut-off titer and its precision) of 57 kits were evaluated using a National Institute of Health Sciences reference standard (NIHS-RS), a pooled serum of COVID-19 patients in a collaborative study.

**Results & discussion**

- The maximum dilution factor of NIHS-RS that gave a positive result differed for each kit, suggesting that the positive judgment criteria, in addition to the limits of detection, differ depending on the kit.
- Concerns were not identified regarding the precision of the results obtained using all kits.
- Exceptionally, some kits for IgM did not produce a positive result even at the highest concentration of NIHS-RS.

**Conclusion**

- A collaborative study using NIHS-RS revealed that antibody assay kits distributed/developed in Japan can generally detect antibodies against SARS-CoV-2.
- Because the characteristics of each kit are different, it is important to use antibody test kits that meet the intended purpose and to interpret the data by understanding the uncertainty of the results.
